# The Association Between Diabetes and Cognition Among Older Hispanics in the United States and Mexicans in Mexico

**DOI:** 10.1093/geroni/igaa057.517

**Published:** 2020-12-16

**Authors:** Jaqueline Avila, Rebeca Wong, Rafael Samper Ternent

**Affiliations:** The University of Texas Medical Branch at Galveston, Galveston, Texas, United States

## Abstract

The objective is to assess if the effect of diabetes on cognition differs by race/ethnicity in the U.S. and how this association differs between older Hispanics in the U.S. and older Mexicans in Mexico. Data comes from a sample of older adults 50 and older with direct interviews from the 2012 waves of the Health and Retirement Study (N=17,810) and the Mexican Health and Aging Study (N=13,270). Cognition was measured as a total cognition score. OLS regressions were used to test the association between diabetes and cognition by race/ethnicity in the U.S. and among older Mexicans in Mexico. Results showed that Non-Hispanic Whites (NHW) had the highest cognition scores in the U.S., followed by Hispanics and non-Hispanic blacks (NHB). Mean cognition score of older Mexicans was higher than for NHB and Hispanics in the U.S. but lower than NHWs. The prevalence of diabetes was highest among Hispanics (32.3%), followed by NHB (30.6%) and NHW (19.9%). The prevalence of diabetes in Mexico was like those NHW in the U.S. (19.9%). In the U.S., the effect of being NHB and Hispanic (compared to white) on cognition was equivalent to having 5.3 and 2.4 fewer years of education, respectively. However, the effect of diabetes on cognition did not differ by race/ethnicity. The final analysis will include a direct comparison between Hispanics in the U.S. and a matched sample of older adults in Mexico with similar sex and age to test differences in the effect of diabetes on cognition between these two samples.

